# Behavior of VOCs and Carbonyl Compounds Emission from Different Types of Wallpapers in Korea

**DOI:** 10.3390/ijerph110404326

**Published:** 2014-04-17

**Authors:** Jungyun Lim, Suejin Kim, ARong Kim, Wooseok Lee, Jinseok Han, Jun-Seok Cha

**Affiliations:** 1 Environmental Infrastructure Research Department, National Institute of Environmental Research (NIER), 42 Hwankyeong-Ro, Seo-gu, Incheon 404-708, Korea; E-Mails: michelle98@korea.kr (J.L.); ksk6250@korea.kr (A.K.); lee8080@korea.kr (W.L.); 2 Environmental Health Research Department, National Institute of Environmental Research (NIER),42 Hwankyeong-Ro, Seo-gu, Incheon 404-708, Korea; E-Mail: suenier@ korea.kr; 3 Climate and Atmospheric Research Department, National Institute of Environmental Research (NIER), 42 Hwankyeong-Ro, Seo-gu, Incheon 404-708, Korea; E-Mail: nierhan@korea.kr

**Keywords:** building materials, wallpaper, volatile organic compounds, carbonyl compounds, small chamber

## Abstract

Emissions of volatile organic compounds (VOCs) and carbonyls from three types of commercially available wallpapers (*i.e.*, PVC-coated, paper-backed, natural material-coated) in Korea were evaluated using a 20 L small chamber. A total of 332 products were tested for emission factors, frequencies of occurrence and composition ratios. Toluene and formaldehyde concentrations were below Korean standard values for all products; however, the total VOC (TVOC) concentrations exceeded current standards (4.0 mg/m^2^·h) for 30 products. The TVOC emission factor for PVC-coated wallpapers, for which polymer materials are used in the manufacturing process, was seven and 16 times higher than those of paper-backed and natural material-coated wallpapers, respectively. The detection frequencies for toluene and formaldehyde were the highest (82.5%) and fourth highest (79.5%), respectively among the 50 target chemical species. The composition ratios for BTEX ranged from 0.3% to 5.1% and unidentified VOCs, which were not qualitatively analyzed using standard gas methods, ranged from 90.2% to 94.8%. Among six carbonyl compounds (acrolein was not detected in any type of wallpaper), acetone had the highest concentrations in PVC-coated (44.6%) and paper-backed (66.6%) wallpapers. Formaldehyde emissions were highest (64.6%) for natural material-coated wallpapers, a result of the formaldehyde-based resin used in the manufacturing process for these products.

## 1. Introduction

Since early 2000, issues related to the sick building syndrome (SBS) and the sick house syndrome (SHS) have been raised in Korea as a result of the deterioration of indoor air quality. The effects of indoor air pollution, which is generated by the continuous circulation of polluted air within a limited space on human health have increased. Although the inflow of polluted outdoor air, human activities and insufficient air circulation in buildings can cause a deterioration in indoor air quality, building materials containing hazardous chemical are the major contributors of indoor air pollution.

Building materials are important sources of volatile organic compounds (VOCs) and formaldehyde in indoor environments. VOCs emitted from building materials are absorbed into the body through respiration and the skin and causes an adverse effects such as eye and respiratory irritations, headaches and asthmatic symptoms. VOCs also have a narcotic influence, suppressing the central nervous system. In particular, benzene is a proven carcinogen that can cause leukemia in those who inhale this compound. In addition, formaldehyde is considered a major driver of SBS and can cause a variety of allergic disorders (e.g., asthma) as an eye and respiratory irritant. The main sources of this compound include pressed-wood products, products made with urea-formaldehyde resins and woven fabrics (e.g., carpets and curtains). Because sufficient evidence exists for carcinogenesis through animal and human testing, formaldehyde has been reclassified from “probably carcinogenic to humans (Group 2A)” to “carcinogenic to humans (Group 1)” by the International Agency for Research on Cancer (IARC, 2006) [1].

The Ministry of Environment of Korea prepared the “Indoor Air Management Act for Public Facilities” in 2004, which established the acceptable standards for total VOC (TVOC), toluene and formaldehyde concentrations emitted from building materials. This act also established a restriction system for the use of building materials that exceed these standards in an effort to improve indoor air quality.

Extensive research has been conducted on VOC emission behaviors from building materials, including emission chamber studies [2,3], perspective/general reviews [4,5] and emission models that predict the behaviors of VOCs and semi volatile organic compounds (SVOCs) [6]. In addition, several papers have reported on the emission of VOCs and formaldehyde from wood-based panel and flooring materials based on their manufacturing processes and test methods [7,8,9,10,11]. Various comparative studies were also performed; these studies include a cooperative analysis of wood-based panels by six European laboratories [12], a comparative study of VOCs in Singapore and European Union office buildings [13], a study that examined emission from carpets using four types of chambers [14] and an analysis of emissions from wood-based panels using 20 L chambers and Field and Laboratory Emission Cell (FLEC) methods [15].

Research on VOC and SVOC emissions from flooring installation materials (e.g., primer, adhesive, floor covering) has also been conducted [16]. A study that examined emissions from laminate flooring as a function of structure and heating condition found that more formaldehyde and TVOC were emitted when buildings used floor heating and air circulation systems, respectively [17,18]. The effects of temperature, preconditioning time and paint coating weight on VOC and formaldehyde emissions have also been investigated [19,20].

VOC emission rates were also measured on-site from a complete structure as well as from individual PVC materials; emissions were significantly higher from the complete structure. [21]. A new Field and Laboratory Emission Cell-Solid Phase Micro Extraction (FLEC-SPME) passive sampling method has been optimized to analyze VOCs from solid building materials [22]. Furthermore, a new method was previously suggested for reducing formaldehyde and VOCs emissions from the resin used to bond the face of fancy veneers in engineered flooring by replacing Urea Formaldehyde (UF) resin with a natural Cashew Nut Shell Liquid (CNSL) [23]. In addition, a new type of eco-friendly wallpaper has been fabricated that use non-metallic minerals, which has decreased the emission ratios for TVOC and formaldehyde compared to those of conventional wallpapers [24].

Although there is extensive research on formaldehyde and VOC emissions from wood-based composites and flooring materials, few studies focused on wallpapers. Unlike many western countries, most Korean homes use wallpaper, and this material occupies the largest surface area in Korean residential environments.

In this study, a 20 L small emission chamber was used to determine levels of VOC and carbonyl emissions from three different types of commercially available wallpapers in Korea. In total, 332 products were tested for major pollutants (*i.e.*, TVOC, toluene and formaldehyde) emission factors, frequencies of occurrence, concentrations and composition ratios of VOCs and carbonyls.

## 2. Experimental Section

### 2.1. Materials

In this study, 332 wallpaper products (Table 1) were analyzed for emission characteristics, all of which were commercially available in the Korean market at the time of study. Based on the manufacturing method, wallpapers were classified as “PVC-coated”, “paper-backed” or “natural material-coated”. PVC-coated wallpapers have a paper substrate with a surface that is coated by heat-softened PVC using plasticizers or metal stabilizers; paper-backed wallpapers are composed of a paper substrate that is laminated to another sheet of paper using an adhesive; and natural material-coated wallpapers are manufactured by coating a mixture of plant leaf materials with mineral powders (e.g., diatomite or illite) on a paper substrate.

The products, which were available to the general public at the time of study, were purchased through a retail dealer within six months of their production to prevent additional external pollution and volatilization through the distribution process. The packaging was not opened until the test samples were prepared and products were stored in the same test conditions (25 °C, 50%–60% relative humidity) away from direct sunlight. Emission tests were performed within 14 days after sample preparation.

**Table 1:** Type and number of wallpaper products tested in this study.

Product Class	Type of Products	Number of Products	Total
Wallpaper	PVC-coated	179	332
	Paper-backed	122	
	Natural material-coated	31	

### 2.2. Sample Preparation

**Figure 1:**
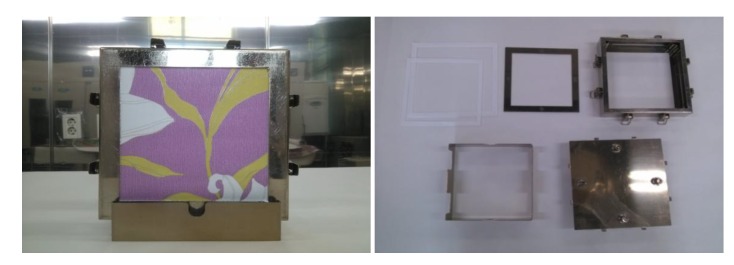
Photograph of an example wallpaper test sample. Parts of specimen holder Installed sample.

Test samples were prepared according to ISO 16000-11 [25,26] and the “Standard Test Method for Indoor Air” by the Ministry of Environment of Korea (Figure 1). The sample length and width were 16.2 cm × 16.2 cm for all products with actual exposure areas of 14.7 cm × 14.7 cm to satisfy a sample loading factor of 2.0 ± 0.2 m^2^/m^3^. Test samples were prepared from wallpaper that was 2 m from the end of the roll (when the roll was longer than 2 m) or from the middle of the roll (when the roll was shorter than 2 m). If there was a pattern in the roll surface, the pattern was set to locate at the middle of the sample.

### 2.3. The 20 L Small Chamber System

**Figure 2:**
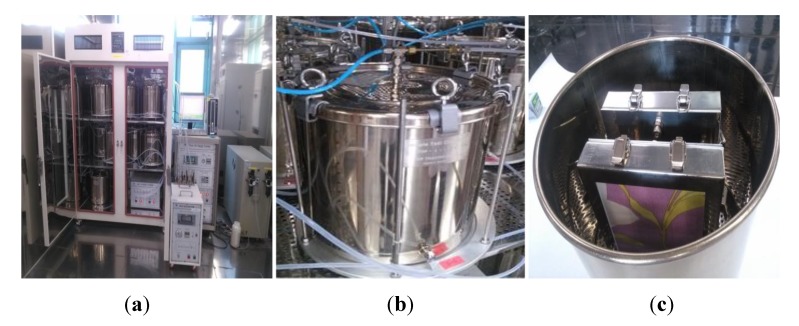
Photograph of the 20 L small emission chamber used in this study. (**a**) Chamber inside; (**b**) Chamber outside; (**c**) Constant T & H system.

The 20 L small chamber method is currently the standard method for testing VOCs and formaldehyde emissions in Korea. The chamber (Top-trading ENG, Seoul, Korea) consisted of distinct systems for air supply, air cleaning, constant T & H (temperature and humidity) maintenance and mass flow control (Figure 2).

The temperature of air supplied to the chamber was held constant using a temperature controlled chamber system. The temperature and relative humidity inside the chamber were maintained at 25 ± 1 °C and 50 ± 5%, respectively. In addition, an air cleaner equipped with 10 stage filters was installed to supply clean air to the chamber. The filter system consisted of a 2-staged silica gel, a 3-staged molecular sieve and a 5-staged activated carbon. A micro-filter (10 μm) was installed at the back of the final activated carbon filter to prevent the inflow of micro-activated carbon particles into the chamber.

### 2.4. Emission Test Conditions

The chamber was composed of stainless steel and treated by electro polishing (EP) to minimize the adsorption of pollutants. The air exchange rate was maintained at 0.5 ± 0.05 h using an automatic mass flow controller and the concentrations of TVOC and formaldehyde in the air supplied to the chamber were maintained below 20 μg/m^3^ and 5 μg/m^3^, respectively. Test conditions within the chamber are summarized in Table 2.

**Table 2:** Test conditions for the 20 L small chamber method used in this study.

Parameter	Condition
Chamber volume	20 L
Chamber material	Stainless-steel (E.P.)
Temperature & humidity	25 ± 1 °C, 50 ± 5%
Specimen area	432.2 cm^2^
Loading factor	2.0 ± 0.2 m^2^/m^3^
Air exchange rate	0.5 ± 0.05 h

### 2.5. Sampling and Analysis

Chamber air was collected 7 days after test specimens were installed using a multi-channel sampling pump that was equipped with a mass flow controller. The amount sampled was below 80% of the total mass flow. VOCs were collected using a solid adsorption tube (Supelco, Bellefonte, PA , USA) packed with Tenax-TA for 25 min at a flow rate of 130 mL/min. Formaldehyde was collected using a 2,4-dinitrophenylhydrazine (DNPH) cartridge (Supelco) equipped with an ozone scrubber for 80 min at a flow rate of 130 mL/min. Most of the adsorbed tubes were analyzed immediately after sampling, tubes that were not immediately analyzed were sealed in a 50 mL vial with a 1/4 inch plug (Swagelok-type with PTFE ferrules) and stored in a refrigerator below 4 °C. All analyses were carried out within 2 weeks of collection. DNPH cartridges were sealed with aluminum foil to protect them from direct sunlight exposure and stored in a refrigerator below 4 °C. The VOC analyses were performed using a thermal desorption unit and a GC/MS, and aldehyde analyses were conducted with an HPLC equipped with a UV-director. The conditions for VOC and formaldehyde analyses are listed in Table 3 and Table 4, respectively. In this study, 44 species, including BTEX, and seven other species, including formaldehyde, acetaldehyde and acetone, were chosen as target VOCs and carbonyl compounds, respectively. TVOC and unidentified compounds were calculated as toluene equivalents.

**Table 3:** Conditions for VOC analysis using the GC/MS.

Thermal Desorber		GC/MS	
**Parameter**	**Condition**	**Parameter**	**Condition**
Instrument	STD 1000, DANI, Italy	Instrument	Shimadzu, GC-2010, Japan
Purge temp. and time	40 °C, 1.0 min	GC column	VB-1 (0.25 mm, 60 m, 1.0 μm)
Desorption time and flow	15 min, 100 mL/min	Split ratio	10:1
Desorption temp.	300 °C	Initial temp.	40 °C (6 min)
Cold trap holding time	15 min	Oven ramp rate 1	4 °C/min (40–180 °C)
Cold trap high temp.	320 °C	Oven ramp rate 2	20 °C/min(180–250 °C)
Cold trap low temp.	−10 °C	Final temp.	250 °C (10 mi.)
Cold trap packing	Tenax-TA	Column flow	1.5 mL/min
Split	No	MS source temp.	200 °C
Valve temp.	210 °C	Detector type	EI (Quadropole)
Transfer line temp.	240 °C	Mass range	35–350 amu
		Electron energy	70 eV

**Table 4:** Conditions for formaldehyde analysis using the HPLC.

Parameter	Condition
Instrument	Waters alliance 2,695 separation module
Column	Waters sunfire C18 (150 mm × 4.6 mm × 3.5 μm)
Detector	Waters 2,487 dual absorbance detector
Mobile phase	Acetonitrile (A)/Water (B)
Gradient elution	0–3 min: A/B = 60/40→60/40 3–8 min: A/B = 60/40→80/20 8–10 min: A/B = 80/20→100/0 10–20 min: A/B= 100/0→60/40
Detection	Absorbance at 360 nm
Mobile phase flow rate	1.0 mL/min
Injection volume	10 μL

### 2.6. Calculation of Emission Factor

The emission factor (*EF*; mg/m^2^·h) was calculated using the following equation:

EF = C_s _(N/L) (1)

where *C_s_* is the equilibrium chamber concentration at a steady-state (mg/m^3^), *N* is the chamber air exchange rate (h^−1^), and *L* is the chamber loading factor (m^2^/m^3^). The air exchange rate was determined by the flow of clean air into the chamber per hour divided by the chamber volume, which indicates the amount of dilution and flushing that occurred within the chamber. The loading factor is the ratio of the total surface area of the materials exposed to the volume of air in the chamber.

## 3. Results and Discussion

### 3.1. Emission Factors of Major Pollutants

In Korea, the Ministry of Environment has promulgated the “Restriction system on the use of pollutant-releasing building materials”, prohibiting the use of materials exceeding established standards in new construction and renovation. Here, we report on the data collected for wallpapers over 3 years. Currently, emission standards exist for three criteria pollutants in Korea, including toluene (0.08 mg/m^2^·h), TVOC (4.0 mg/m^2^·h), and formaldehyde (0.12 mg/m^2^·h). Countries having the emission standards for wallpaper are very few because the wallpaper is not popularly used in most western countries. However, in Finland, the emission standards of VOC and formaldehyde for all type of building materials (brick, ceramic tile, board *etc.*) are 0.4 and 0.125 mg/m^2^·h, respectively. The standard for formaldehyde is similar in two countries, but the standard for VOC is much tighter in Finland since it is under certification system in that country. Of the 332 tested products, 30 exceeded the current standards for TVOC, however, no products exceeded the standards for toluene and formaldehyde. The emission factors for TVOC for the 30 products that exceeded standards ranged from 4.528 to 13.748 mg/m^2^·h, with a mean emission factor of 7.785 mg/m^2^·h.

**Figure 3:**
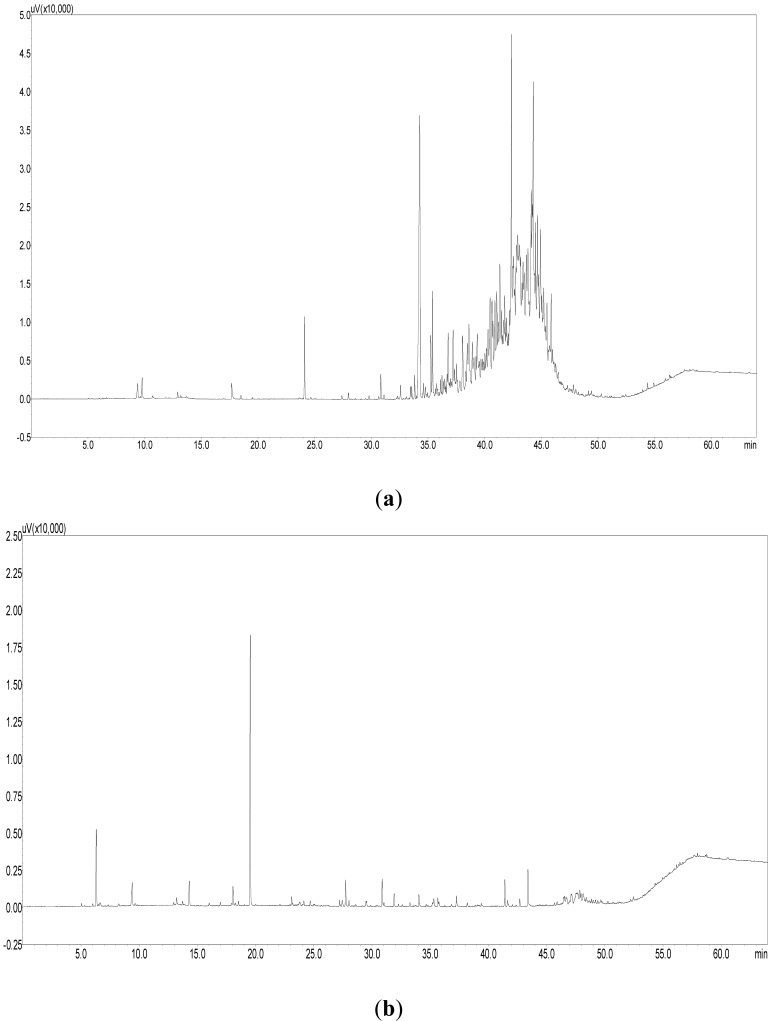
Chromatogram for the volatile organic compounds emitted from different types of wallpapers. (**a**) PVC-coated wallpaper, (**b**) Non-PVC-coated wallpaper.

Table 5 shows the emission factors for TVOC, toluene and formaldehyde for the three types of wallpapers. The mean TVOC emission factor for PVC-coated wallpapers was 2.146 mg/m^2^h, which was seven and 16 times higher than that of paper-backed (0.304 mg/m^2^h) and natural material-coated wallpapers (0.137 mg/m^2^·h). The GC/FID chromatograms for VOCs from PVC-coated and non-PVC-coated wallpapers are shown in Figure 3. Various polymer materials were detected in the chromatogram from PVC-coated wallpapers, and the shapes of the peaks were very different from those of non-PVC-coated wallpapers. Because PVC-coated wallpaper is manufactured by coating the paper substrate with heat-softened PVC using plasticizers or metal stabilizers, it is reasonable to assume that various polymer peaks came from the raw input materials that were part of the manufacturing process. Generally, the decane (C_10_) class comprised more than 90% of TVOC emissions even though there was a slight difference by the products.

Sarigiannis *et al.* performed a comprehensive exposure study to VOCs and carbonyls in European indoor environments. The typical concentrations of toluene in residences or houses ranged from 4–5 to 30 µg/m^3^, although concentrations as high as 80 µg/m^3 ^have been registered [27]. In our study, the mean emission factor of toluene was estimated as 0.0081 mg/m^2^∙h (Table 6) which is corresponding to 35.6 µg/m^3^. Although this work was done by chamber study, the concentration level of toluene is in the comparable range with EU. In the case of formaldehyde, Sarigiannis *et al.* report typical indoor formaldehyde concentrations ranging between 10 and 50 µg/m^3^ which is also comparable with our result (17.6 µg/m^3^). The emission rates for toluene and, in particular, formaldehyde were generally low for all types of wallpapers, with the emission factors for formaldehyde being orders of magnitude below the standard. However, the mean emission factor for formaldehyde for natural material-coated wallpapers was about 1.6 and 4.0 times higher than that of the PVC-coated and paper-backed wallpapers, respectively. Most natural material-coated wallpapers are manufactured by attaching woodchips or plant leaf materials directly to the wallpaper or by injecting/coating a powdered version of these materials to the wallpaper. Formaldehyde-based resin binders are generally used in this process, and the high emission levels for formaldehyde could be attributed to the use of these resins [7].

**Table 5:** A summary of pollutant emission factors from three types of wallpapers.

Material	Pollutant	Emission Factor (mg/m^2^·h)			SD
		Mean	Min	Max	
PVC-coated (n = 179)	TVOC	2.146	0.017	13.748	2.875
	Toluene	0.003	-	0.066	0.006
	Formaldehyde	0.005	-	0.094	0.010
Paper-backed (n = 122)	TVOC	0.304	0.001	6.896	0.852
	Toluene	0.013	-	0.181	0.026
	Formaldehyde	0.002	-	0.024	0.004
Natural material-coated (n = 31)	TVOC	0.137	0.002	0.822	0.176
	Toluene	0.004	-	0.028	0.007
	Formaldehyde	0.008	-	0.040	0.012

**Table 6:** Frequencies of occurrence and emission factors for chemicals evaluated in this study.

Frequency of Occurrence				Emission Factor (mg/m^2^·h)			
Rank	Chemical	No. of Products	% of Products	Chemical	Mean	Max	Min
1	Toluene	274	82.5	*n*-Tridecane	0.0161	1.3362	-
2	*n*-Undecane	266	80.1	*n*-Tetradecane	0.0141	0.1853	-
3	*n*-Tetradecane	265	79.8	Acetone	0.0131	2.2741	-
4	Formaldehyde	264	79.5	Decanal	0.0082	0.2572	-
5	*o*-Xylene	263	79.2	Toluene	0.0081	0.2263	-
6	1,2,4-Trimethyl-benzene	259	78.0	*n*-Pentadecane	0.0062	0.0625	-
7	*n*-Dodecane	257	77.4	Formaldehyde	0.0040	0.0944	-
8	Ethylbenzene	256	77.1	*n*-Dodecane	0.0039	0.0624	-
9	*m,p*-Xylene	255	76.8	*n*-Undecane	0.0037	0.0500	-
10	*n*-Tridecane	248	74.7	Acetaldehyde	0.0036	0.5799	-

### 3.2. Frequencies of Occurrence and Concentrations of Chemicals

Detection frequencies and emission factors for the top 10 most prevalent chemicals (among 50:43 VOCs and seven carbonyls) are presented in Table 6.

Toluene, found in 274 products (82.5%), was the most frequently detected chemical. The mean emission factor for toluene was the fifth highest of the target chemicals. Moreover, aromatic VOCs, such as xylene and ethylbenzene, as well as toluene were detected in more than 75% of the products. The chemical components of various plasticizers, diluents and inks used in wallpaper manufacturing and printing processes are major contributors of these emission levels. Formaldehyde was detected in 264 products (79.5%; fourth highest detection frequency), with a mean emission factor of 0.004 mg/m2·h (seventh highest among all chemicals). The emission factors were of the order *n*-tridecane > *n*-tetradecane > acetone > decanal > toluene, and the decane class generally had a higher frequency of occurrence and emission factors. This bias occurred because we tested a disproportionately higher number of PVC-coated products which emit a large quantity of pollutants above decane.

### 3.3. Composition Ratios for VOCs and Carbonyls

**Figure 4:**
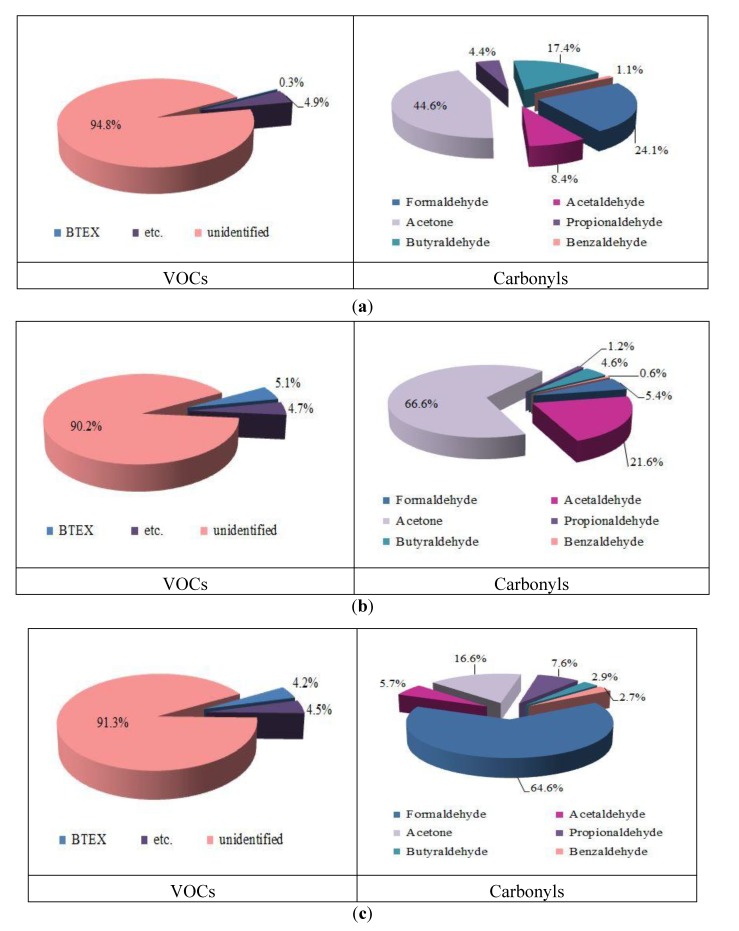
Composition ratio for VOCs and carbonyls from three types of wallpapers. (**a**) PVC-coated; (**b**) Paper-backed; (**c**) Natural material-coated.

Figure 4 shows the composition ratios for VOCs and carbonyls for the three types of wallpapers analyzed. In all three groups, unidentified VOCs accounted for more than 90% of all VOCs. This was because most VOCs were not qualitatively detected by the standard gases containing 44 species we have used.

For the PVC-coated wallpapers, TVOC was largely comprised of unidentified VOCs (94.8%). In addition, BTEX comprised only a small percentage of TVOC (0.3%), illustrating that the contribution of these major VOCs was very small. In paper-backed and natural material-coated wallpapers, the unidentified VOCs comprised 90.2% and 91.3% of TVOC, respectively, and the proportions of BTEX were 5.1% and 4.2%, respectively. The BTEX values were 17 and 14 times higher than PVC-coated wallpapers for the paper-backed and natural material-coated wallpapers, respectively. As shown in the chromatogram for PVC-coated wallpapers (Figure 3), VOCs detected from hexane (C_6_) to hexadecane (C_16_) were very diverse, while the proportions of decane (C_10_) were very high compared to that of non- PVC-coated wallpapers. The raw materials used in the manufacturing process for PVC-coated wallpaper primarily include high-carbon-numbered chemicals compared to the processes for paper-backed and natural material-coated wallpapers, likely resulting in the lower concentration of BTEX for the PVC-coated wallpapers [7]. The proportions of VOCs that can be analyzed qualitatively (noted as “*etc*.” in Figure 4), excluding the unidentified VOCs and BTEX, were similar (4.5%–4.9%) for all three types of wallpapers.

The carbonyl concentrations differed among the types of wallpapers, and acrolein was not detected in any type. Among the six carbonyl compounds the proportion of acetone was highest for PVC-coated (44.6%) and paper-backed (66.6%) wallpapers. The high acetone emission levels from PVC-coated and paper-backed wallpapers were likely due to the inks used as solvents for printing patterns or colors in the manufacturing processes, of which acetone is a major component [7]. In addition, the emission of butyraldehyde was especially high for PVC-coated wallpapers compared to other wallpapers. This result was likely because DEHP (diethylhexylphthalate) is normally used as a plasticizer in the manufacturing process for PVC-coated wallpapers and butyraldehyde is also used as a raw material in making DEHP plasticizer. The formaldehyde emission rates were generally very small for all 3 types of wallpapers. The proportion of this compound was highest (64.6%) for natural material-coated wallpapers, likely because phenol-formaldehyde (PF) resins are used as an adhesive for binding paper and natural materials.

## 4. Conclusions

The emission characteristics for VOCs and carbonyls for the three different types of wallpapers (332 total products), currently available in Korea, were examined. Results showed that toluene and formaldehyde emissions did not exceed the established standards for any products; however, 9% of the products exceeded the standards for TVOC. PVC-coated wallpapers had a mean TVOC emission factor that was seven and 16 times higher than that of paper-backed and natural material-coated wallpapers, respectively. Toluene was detected in 274 products (82.5%) and had the highest frequency of occurrence. Formaldehyde had the fourth highest detection frequency (79.5%) and the seventh highest mean emission factor. Unidentified VOCs comprised 90.2%–94.8% of the TVOC emissions. BTEX emissions for paper-backed and natural material-coated wallpapers were 17 and 14 times higher than that of PVC-coated wallpapers, although these emissions only ranged from 0.3% to 5.1%. Among the six carbonyl compounds evaluated (acrolein was not detected in any type of wallpapers), acetone was most common in PVC-coated (44.6%) and paper-backed (66.6%) wallpapers. Formaldehyde emissions were highest for natural material-coated wallpapers (64.6%) out of the six carbonyl compounds, which was due to the formaldehyde-based adhesive resins used in the manufacturing processes for these products.
